# Extracellular vesicle signatures and protein citrullination are modified in shore crabs (*Carcinus maenas*) infected with *Hematodinium* sp

**DOI:** 10.1080/21505594.2023.2180932

**Published:** 2023-03-02

**Authors:** Christopher J. Coates, Igor Kraev, Andrew F. Rowley, Sigrun Lange

**Affiliations:** a Department of Biosciences, College of Science, Swansea University, Swansea, UK; b Zoology, Ryan Institute, School of Natural Sciences, University of Galway, Galway, Ireland; c Electron Microscopy Suite, Faculty of Science, Technology, Engineering and Mathematics, The Open University, Milton Keynes, UK; d Tissue Architecture and Regeneration Research Group, School of Life Sciences, College of Liberal Arts and Sciences, University of Westminster, London, UK

**Keywords:** Peptidylarginine deiminase (PAD), arginine deiminase (ADI), innate immunity, haemocytes, cell-cell communication, marine disease

## Abstract

Epizootiologists recurrently encounter symbionts and pathobionts in the haemolymph (blood equivalent) of shellfish. One such group is the dinoflagellate genus *Hematodinium*, which contains several species that cause debilitating disease in decapod crustaceans. The shore crab *Carcinus maenas* acts as a mobile reservoir of microparasites, including *Hematodinium* sp., thereby posing a risk to other co-located commercially important species, e.g. velvet crabs (*Necora puber*). Despite the widespread prevalence and documented seasonality of *Hematodinium* infection dynamics, there is a knowledge gap regarding host-pathogen antibiosis, namely, how *Hematodinium* avoids the host’s immune defences. Herein, we interrogated the haemolymph of *Hematodinium*-positive and *Hematodinium*-negative crabs for extracellular vesicle (EV) profiles (a proxy for cellular communication), alongside proteomic signatures for post-translational citrullination/deimination performed by arginine deiminases, which can infer a pathologic state. Circulating EV numbers in parasitized crab haemolymph were reduced significantly, accompanied by smaller EV modal size profiles (albeit non-significantly) when compared to *Hematodinium*-negative controls. Differences were observed for citrullinated/deiminated target proteins in the haemolymph between the parasitized and control crabs, with fewer hits identified overall in the former. Three deiminated proteins specific to parasitized crab haemolymph were actin, Down syndrome cell adhesion molecule (DSCAM), and nitric oxide synthase – factors that contribute to innate immunity. We report, for the first time, *Hematodinium* sp. could interfere with EV biogenesis, and that protein deimination is a putative mechanism of immune-modulation in crustacean-*Hematodinium* interactions.

## Introduction

The common (green) shore crab *Carcinas maenas* is native to European coastal waters, yet represents one of the most damaging marine invasive species having extended its non-native range to the Americas, Australia, and Asia [1,2]. In part, ecosystem changes are brought about by *C. maenas* and their attendant pathogens and parasites. These crabs are mobile reservoirs of diverse symbionts and pathobionts [3–5], including the parasitic dinoflagellate genus *Hematodinium* [6,7], and taxa with zoonotic potential towards bivalves, such as, ostreid herpesvirus [8] and *Haplosporidium* spp [9]. *Hematodinium* spp. are haemolymph-borne parasites that have blighted ~50 free-roaming and captive decapod species globally [10–12], including lucrative fisheries: Atlantic blue crabs (*Callinectes sapidus* [13,14], langoustine (*Nephrops norvegicus* [15–17], edible crabs (*Cancer pagurus* [18,19], snow and tanner crabs (*Chionoecetes* spp [20,21].

*Hematodinium* spp. have been designated an emerging disease in crab polyculture systems, e.g. ridgetail white prawns (*Exopalaemon carinicauda* [22]; and the gazami crab (*Portunus trituberculatus* [23], which is concerning given the absence of control methods to limit *Hematodinium* spread (reviewed by [24]). Enhancing our understanding of the crab’s immune response or dysregulation during *Hematodinium* sp. infection is essential for developing strategies that can disrupt the transmission cycle or treat diseased animals. We know much about the immunocompetence of decapod crustaceans toward bacteria, fungi, and viruses, including specific antimicrobial factors like carcinin and phenoloxidase enzymes [25–29], but there are fewer studies concerned with how *Hematodinium* spp. influence crab immunity [30]. For gazami crabs (*P. trituberculatus*) infected with *H. perezi*, Li et al. [31,32] applied multi-omic approaches to the hepatopancreas and haemolymph (containing haemocytes), revealing potential immune-suppressive strategies of the dinoflagellate in a bid to colonize the host, such as, disrupting phagosome formation. To the best of our knowledge, no *Hematodinium*-derived factor has been identified to date with a direct immune-suppressive role.

For our shore crab-*Hematodinium* sp. pathosystem, we sought further insight into the haemolymph state at the cellular and protein levels by taking inspiration from chordate systems – assessing extracellular vesicle traffic during inflammation and haemolymph post-translation signatures with an emphasis on citrullinated/deiminated proteins. Peptidyl-arginine deiminases (PADs) represent an enzyme family in the Chordata, which catalyse post-translational modification of arginine to citrulline (citrullination/deimination) in proteins, affecting protein structure and function; linked to pathobiological processes through the generation of neo-epitopes, changes in protein–protein interactions, gene regulation, and extracellular trap formation (ETosis). PAD homologues, namely arginine deiminases (ADIs), were also identified in several pathogens, including bacteria, fungi, and macroparasites [33–37], and their deiminating activity on host proteins has been implicated in host-tissue remodelling and pathogen immune-evasion [33,36,38], including *via* extracellular vesicle (EV) modulation [35,36]. EVs are circulating membrane blebs that play key roles in cellular communication, and are released from cells in body fluids, and carry a range of proteomic, DNA, and non-coding RNA species cargo, which can influence cellular communications. Changes in systemic EV responses are indicative of pathology and immunity, often observed in raised EV numbers in response to harmful stimuli and changes in EV cargo content. Extracellular vesicle profiles, as well as citrullinated proteins in healthy marine invertebrates have been reported for biomedically important Atlantic horseshoe crabs (*Limulus polyphemus* [39], fished American lobsters (*Homarus americanus* [40], several cultured bivalves [41] and purple sea urchins (*Strongylocentrotus purpuratus* [42], but not for shore crabs. Magnadóttir et al. [43], described changes in EV signatures in reared fish (Atlantic cod, *Gadus morhua*) in response to environmental temperature, highlighting their biomarker potential.

Our aim was to determine whether cellular (haemocyte) communication was compromised in crabs during patent *Hematodinium* sp. infection, via extracellular vesicle (EV) or deimination mediated pathways. To address this aim, we (1) compared EV profiles in the haemolymph of *Hematodinium-*positive and -negative crabs, and (2) used proteomics in conjunction with immunoprecipitation to account for signatures of post-translational modification of immune factors (i.e. deimination/citrullination).

## Materials and methods

### Shore crabs and haemolymph inspection

**Figure 1.**
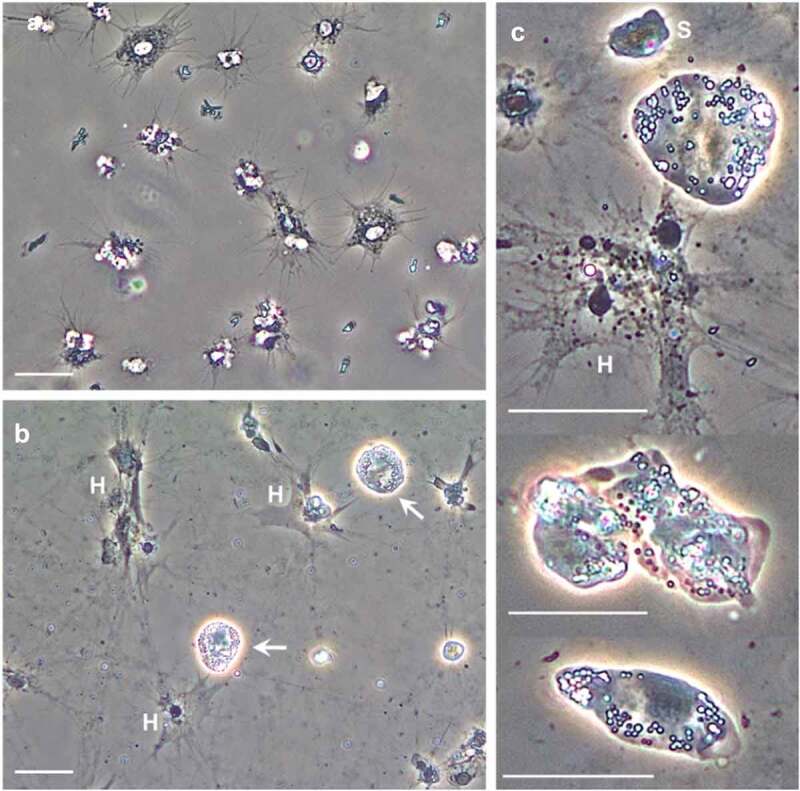
*Hematodinium* sp. morphotypes in the haemolymph of shore crabs, *Carcinus maenas*. Freshly withdrawn haemolymph was inspected using phase contrast microscopy. a) Appearance of haemolymph absent *Hematodinium* sp. b) *Hematodinium* sp. (white arrows) are highly refractile compared to shore crab haemocytes (H). When in contact with a surface, the haemocytes settle, spread, and lose their refractile properties. c) Higher magnification views of *Hematodinium* sp. variation; small (S) and large uninucleate, irregular and elongate shapes. Scale bars represent 20 µm (a, b) and 25 µm (c).

*Carcinus maenas* (*n* = 58) were collected using baited pots immersed in the Prince of Wales Dock, Swansea Bay (Wales, UK) in July 2021. Crab carapace width (mm) was measured with a Vernier callipers, and biometric data including moult stage (inter- or post-moult) sex (male, female), missing/damaged limbs, presence/absence of fouling or ectoparasites, and shell disease were recorded. Haemolymph (~550 µL) was accessed by inserting a 22-gauge hypodermic needle attached to a sterile syringe through the arthrodial membrane of a walking leg. For each crab, two haemolymph aliquots of ~100 µL and~300 µL (including haemocytes) were placed into separate sterile micro-centrifuge tubes and frozen immediately at −70°C for subsequent molecular work (DNA extraction, PCR), extracellular vesicle (EV) and proteomic measurements, respectively. An additional 25 µL of freshly withdrawn haemolymph was placed onto a glass slide for inspection of known microparasites via microscopy (e.g. yeast-like fungi, *Haplosporidium* spp.). Haemocytes were allowed to settle/adhere onto the glass slide for~10 minutes prior to inspection under phase contrast settings using an Olympus B×41 microscope. *Hematodinium* sp. presence was confirmed based on their appearance – the parasites retain their refractivity and do not spread (Figure 1(a,b)). Within a field of view, the ratio of *Hematodinium* to haemocytes was recorded. Finally, ~100 µL haemolymph was added to an equal volume of sterile 3% NaCl (w/v) solution and spread onto tryptone soya agar (TSA) plates (prepared with an additional 2% NaCl) to determine if cultivable bacterial colony forming units (CFUs) were present. TSA plates were incubated at 25°C for 48 h prior to CFU enumeration.

Genomic DNA was extracted from the haemolymph of six *Hematodinium*-positive crabs (3 male, 3 female) and six apparent *Hematodinium*-negative crabs (3 male, 3 female) and screened for subclinical *Hematodinium* spp. infection as described in [6,7]. Briefly, genomic DNA (~3 µg per reaction) was amplified using validated PCR oligonucleotides (Hemat-For-1487 and Hemat-Rev-1654) targeting the 18S rRNA subunit [44] and thermo-cycling conditions: initial denaturation for 10 min at 94°C, followed by 30 rounds of 94°C for 15 s, 54°C for 15 s, and 72°C for 30 s, and a final elongation step of 72 °C for 10 min.

### Extracellular vesicle isolation and characterization (nanoparticle tracking, Western blotting, transmission electron microscopy)

Extracellular vesicles (EVs) were isolated from the haemolymph of *Hematodinium*-positive (*n* = 6) and -negative (*n* = 6) crabs. Three samples were pooled to achieve ~900 µL for each group (parasitized and non-parasitized males/females). For EV isolation, differential centrifugation was performed as described by Bowden et al. [39]. Per group, 100 µL of haemolymph was added to 400 µL Dulbecco’s Phosphate Buffered Saline (DPBS), centrifuged at 4000 *x* g for 30 min, the supernatant was collected and re-centrifuged at 100,000 *x* g for 1 h at 4°C for retrieval of total EVs. The EV enriched pellet was resuspended in 500 µL DPBS and centrifuged again at 100,000 *x* g for 1 h at 4°C, discarding the supernatant and diluting the EV pellet in 100 µL DPBS. For EV quantification by nanoparticle tracking analysis (NTA), 10 µL of diluted EV pellet was added to 990 µL DPBS and applied onto an NS300 Nanosight (Malvern, UK) using a syringe pump at a flow rate 50. Particles were recorded at camera level 13 with four-times 1 min recordings, and post-analysis was carried out at threshold level 3 with 30–40 particles per window. The four readings were averaged per sample, using the in-built NTA software (version 3, Malvern, UK). EVs were further analyzed by Western blotting for two surface markers, CD63 (ab68418, Abcam, 1/1000) and Flotillin-1 (ab41927, Abcam 1/1000), as well as visualized for morphological assessment using transmission electron microscopy (TEM) as described in Bowden et al. [39] in accordance with the minimum requirements for EV characterization set by the International Society for Extracellular Vesicle Research [45].

### Western blotting

For SDS-PAGE and Western blotting, a 100 µL aliquot per sample was diluted with 100 µL 2 × reducing Laemmli sample buffer (containing 5% [v/v] β-mercaptoethanol), boiled for 5 min at 100°C and a 5 µL aliquot per sample was applied to 4–20% Tris-glycine (TGX^TM^) gels (BioRad, UK). Electrophoresis was carried out at 165 V for 52 min; gels were then transferred for Western blotting analysis using semi-dry transfer (1 h at 15 V), even protein transfer was assessed by PonceauS red stain (Sigma, UK). The membranes were blocked in 5% [w/v] bovine serum albumin (BSA, Sigma, UK) in TBS-T for 1 h at room temperature and incubated in primary antibodies overnight at 4°C on a shaking platform. Primary antibodies used for haemolymph were: anti-PAD2 (ab50257), as this is the most phylogenetically conserved PAD isozyme, and anti-citrullinated histone H3 (citH3, ab5103) as a marker for ETosis assessment; both diluted 1/1000 in TBS-T. For assessment of EV surface markers, CD63 and Flotillin 1 were used as described above. Following primary antibody incubation, the blots were washed with TBS-T (3 × 10 min), incubated in secondary antibody for 1 h at room temperature (using HRP-labelled anti-rabbit IgG; BioRad, diluted 1/3000 in TBS-T). Following washing (5 × 10 min in TBS-T), visualization was carried out using ECL (Amersham Biosciences) and the UVP BioDoc-ITTM System (ThermoFisher Scientific, Dartford, UK). Protein densitometry analysis was carried out in ImageJ [46].

### F95-enrichment for deiminated/citrullinated proteins from haemolymph

For identification of putative deiminated/citrullinated proteins in crab haemolymph, enrichment was carried out using the F95 pan-citrulline antibody (MABN328, Merck) in conjunction with the Catch-and-Release Immunoprecipitation Kit (Merck). Immunoprecipitation was carried out on mini-agarose columns together with the F95 antibody and the affinity ligand, overnight at 4°C on a rotating platform and proteins thereafter eluted according to the manufacturer’s instructions (Merck, UK). F95-enriched proteins from the parasitized and control haemolymph were then subjected either to SDS-PAGE and silver staining, or to liquid chromatography tandem mass spectrometry (LC-MS/MS) analysis for identification of protein hits.

### Silver staining

F95-enriched protein fractions from haemolymph were diluted 1:1 in 2 × reducing Laemmli sample buffer, boiled for 5 min at 100°C and separated on 4–20% TGX gels (BioRad) for 52 min at 165 V. For silver staining, the BioRad Silver stain kit was used, according to the manufacturer’s instructions.

### LC-MS/MS proteomic analysis

For identification of the F95 enriched proteins from crab haemolymph, in-gel digestion was used for LC-MS/MS analysis. Samples were first prepared 1:1 in reducing Laemmli sample buffer, boiled and run 0.5 cm into a 10% TGX gel (BioRad) and then cut out as one whole band per sample (F95-enriched proteins for 2 samples of control haemolymph, containing a pool of 3 individuals each (3 × male, 3 × female), and 2 samples of infected haemolymph, containing a pool of 3 individuals each (3 × male, 3 × female)). Proteomic analysis was carried out by Cambridge Proteomics (Cambridge, UK) according to previously described methods [39], and hits were assessed against the shore crab database CCP_ *Carcinus maenas* _ 20220314 (252 sequences; 58963 residues). For quality control, a common contaminant database was also searched (cRAP 20190401; 125 sequences; 41129 residues). Protein scores were derived from ion scores as a non-probabilistic basis for ranking protein hits; individual ion scores ≥20 indicated identity or extensive homology (*P* < 0.05).

### Data handling

Un-paired t-tests were used to compare EV datasets (numbers and modal size) between parasitized and control crabs, in Graph Pad Prism v7. Statistical significance was regarded as *P* < 0.05. NTA analysis was carried out using the in-built software (v3) and is based on four reads per pooled sample and presented as average reads (black line) with standard error (red line).

## Results

### *Screening shore crab haemolymph for* Hematodinium *sp.*

First, haemolymph from 58 crabs (20 males, 38 females) was viewed using phase contrast settings, and revealed 15.5% infected with *Hematodinium* sp. morphotypes (Figure 1(a–c)) found recurrently in *C. maenas*, and representing trophonts. The ratio of *Hematodinium* to host haemocytes was 1: 22 ± 3.7 (*n* = 6) for the affected crabs, which indicated a low to moderate disease burden. Aside from *Hematodinium*, we found no evidence of sepsis, mycosis, or other haemolymph-borne microparasites known to infect *C. maenas*. Second, end-point PCR with oligonucleotide primers specific for *Hematodinium* spp. did not yield any parasite signals, i.e. amplicons of the expected size (187 bp), in those crabs considered *Hematodinium*-negative from microscopic examination of the liquid tissue. There was no evidence of sub-patent infection. Hence, haemolymph from six parasitized and six healthy crabs (listed in Supplementary Table S1) with mean (± SE) carapace widths of 55.3 ± 2.1 mm and 56.7 ± 2.1 mm, respectively, were pooled for further experimentation. Notably, bacterial CFU levels were negligible for both control (3 out of 6 testing positive with~107 CFUs mL^−1^) and parasitized (2 out of 6 testing positive with~130 CFUs mL^−1^) crabs.

### Extracellular vesicle profiles and citrullinated/deiminated proteins differ between parasitized and control crab haemolymph

**Figure 2.**
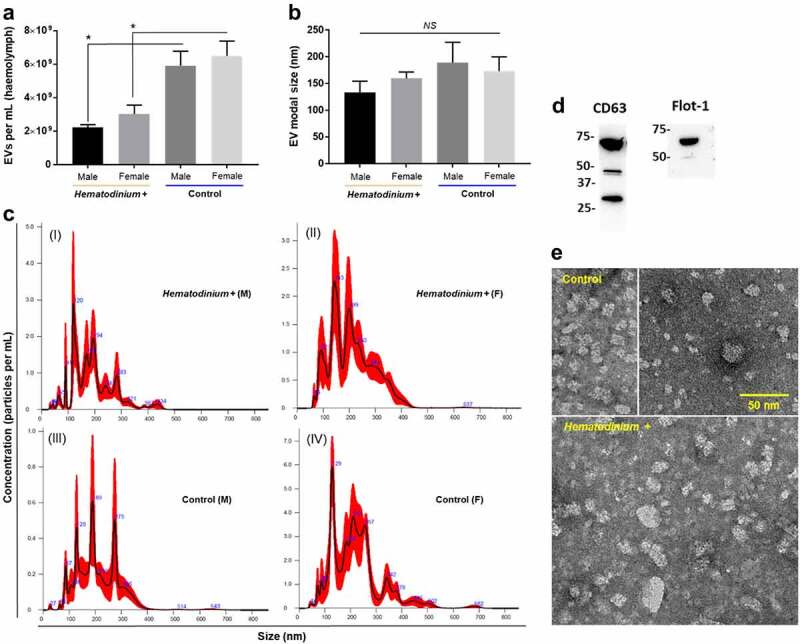
Extracellular vesicle profiles of haemolymph from parasitized and healthy shore crabs. a) Total numbers of EVs quantified in *Hematodinium*-positive and – negative (control) crabs. An asterisk (*) denotes a significant difference (*P* < 0.05). b) Modal size of EVs found in crab haemolymph. NS, non-significant. c) Representative nanoparticle tracking analysis (NTA) curves of haemolymph from parasitized (I, II) and healthy (III, IV) crabs. d) Western blot of pooled crab samples (*n* = 12) used to identify EV-specific markers, namely CD63 (for small EVs, “exosomes”) and Flotillin 1 (Flot-1, for medium EVs, “microvesicles”). e) Representative transmission electron micrographs of haemolymph EVs isolated from control (upper images) and *Hematodinium*-positive (lower image) crabs. Scale bar = 50 nm.

Haemolymph from *Hematodinium*-positive and -negative (healthy) crabs was profiled by nanoparticle tracking analysis (NTA) to assess changes in total extracellular vesicle (EV) numbers, as well as modal size (Figure 2(a–c)). Overall, EV numbers were significantly lower in the haemolymph of parasitized crabs (*P* = 0.0056, H+ male *vs*. H – male; *P* = 0.0163, H+ female *vs*. H – female), ~2.6 × 10^9^ mL^−1^, than healthy crabs 6.2 × 10^9^ mL^−1^, and consistent across both sexes in each group (Figure 2a). The modal size of EVs was smaller in parasitized crabs, 146.5 nm, compared to healthy ones, 181.3 nm (Figure 2(b–c)), albeit the differences were non-significant (*P* > 0.05 in all comparisons). Haemolymph EVs ranged in size from 30 nm to 500 nm, and were positive for both CD63 and Flotillin 1 (Figure 2d), which are key surface markers for small EVs (“exosomes,” CD63) and medium-large sized EVs (“microvesicles,” Flot-1). Transmission electron microscopy (TEM) revealed heterogeneous EV populations in both healthy and parasitized crab haemolymph (Figure 2e).

**Figure 3.**
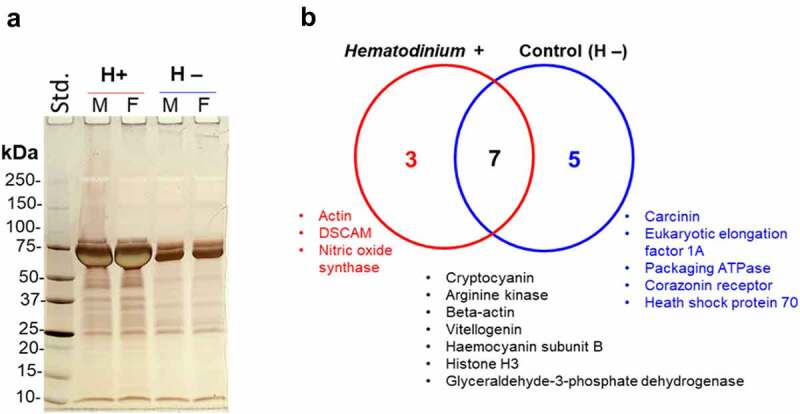
Deiminated/Citrullinated proteins differ in parasitized and control crabs. a) Silver-stained gel showing F95-enriched fractions from the haemolymph of crabs infected with *Hematodinium* sp. and healthy (control). Std, protein standards (ladder); M, male; F, female. b) Venn diagram representing deiminated/citrullinated protein targets specific to, and shared among, parasitized and control crabs, based on proteomic hits with a shore crab database.

F95-enriched proteins from *Hematodinium*-positive and -negative (control) crabs were assessed by silver staining (Figure 3a) and subjected to LC-MS/MS analysis for hits with the shore crab (*C. maenas*) database, revealing eight discrete target proteins of deimination between each group, and a further seven modified proteins common to both groups (Figure 3b) – listed in Table 1.

**Table 1.** F95-enriched protein hits (deiminated/citrullinated) from the haemolymph of *Hematodinium* sp. infected (a) and control (b) crabs. Proteins unique to either parasitized or control crabs are in bold. This list was compiled using searches for proteins restricted to the shore crab (*C. maenas*) database only.

Protein name	Protein ID	Score^a^
(a) *Hematodinium*-positive crabs		
Cryptocyanin (fragment)	A0A6J3WT23_CARMA	2804
Beta-actin (fragment)	A0A346FQ23_CARMA	239
Arginine kinase (fragment)	B3TNF1_CARMA	195
Vitellogenin (fragment)	C6KI51_CARMA	129
Hemocyanin subunit B (Fragment)	P83178|HCYB_CARMA	121
Glyceraldehyde-3-phosphate dehydrogenase (fragment)	A0A346FQ25_CARMA	92
Histone H3 (fragment)	B8ZJ27_CARMA	75
**Actin (fragment)**	**P80709|ACT_CARMA**	**42**
**DSCAM (tail-less isoform)**	**A0A024K6I7_CARMA**	**30**
**Nitric oxide synthase**	**D5I3H5_CARMA**	**20**
(b) *Hematodinium*-negative (control) crabs		
Cryptocyanin (Fragment)	A0A6J3WT23_CARMA	1969
Arginine kinase	Q9U9J4|KARG_CARMA	319
Beta-actin (Fragment)	A0A346FQ23_CARMA	188
Hemocyanin subunit B (Fragment)	P83178|HCYB_CARMA	161
Glyceraldehyde-3-phosphate dehydrogenase (Fragment)	A0A346FQ25_CARMA	160
Vitellogenin (Fragment)	C6KI51_CARMA	153
Histone H3 (Fragment)	B8ZJ27_CARMA	70
**Heat shock protein 70 kDa (Fragment)**	**A0A1B1FGX7_CARMA**	**51**
**Carcinin**	**Q8WQ91_CARMA**	**35**
**Eukaryotic elongation factor 1A (Fragment)**	**A0A346FQ26_CARMA**	**24**
**Packaging ATPase**	**A0A6G9HDY9_9VIRU**	**24**
**Corazonin receptor**	**A0A2L0WQE5_CARMA**	**22**

^a^Protein scores were derived from ion scores as a non-probabilistic basis for ranking protein hits; scores ≥20 indicated identity or extensive homology (*P* < 0.05). Each value represents an average of pooled samples (for both parasitized and control crabs).

### Assessment of putative PAD/ADI and histone H3 citrullination in haemolymph

**Figure 4.**
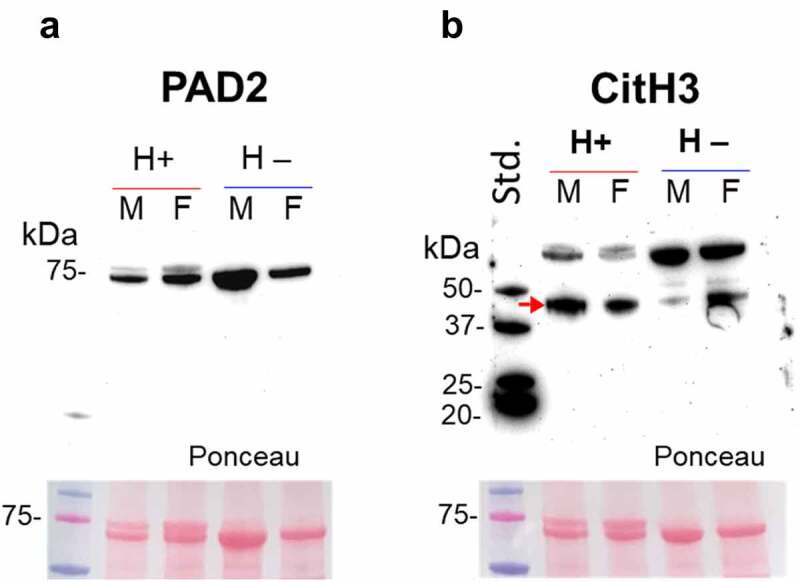
PAD-homologue/ADI assessment and evidence for histone H3 citrullination in shore crabs. a) Using anti-human PAD2 antibody on crab haemolymph, a band at the expected ~75 kDa was observed in both parasitised (males/females) and control (male/female) groups. b) Evidence for histone H3 citrullination, albeit bands are at a slightly higher kDa level than expected. Some differences are observed in the lower band in the parasitised (H+) group (increased signal, indicated by a red arrow) and accompanied by a decrease in the higher band (higher band in the control; H –). Haemolymph. Std., protein standards (ladder); M, male; F, female.

*Hematodinium*-positive and -negative (control) haemolymph was assessed for the presence of ADI (PAD homologue) using the anti-human PAD2 antibody, as PAD2 is considered the phylogenetically most conserved isozyme. Haemolymph was further assessed for the presence of histone H3 citrullination, using the CitH3 antibody, which is also used as a marker for extracellular trap formation (ETosis). A positive band at the expected size of PAD/ADI was observed around 75 kDa size (Figure 4a), while citH3 was observed at higher levels (40 and 70 kDa) than the expected 17 kDa (Figure 4b). Using LC-MS/MS, a histone H3 fragment was identified as deiminated in both parasitized and control crabs (Table 1). Based on Western blotting, a decrease in the intensity of the higher 70 kDa band was observed in the parasitized crabs, accompanied by an increase in the lower band (~40 kDa), which was more marked in the parasitized groups, indicating increased ETosis.

## Discussion

Herein, we aimed to enhance our understanding of the putative mechanisms *Hematodinium* sp. employs to modulate the immune defences of shore crabs, *C. maenas*. We performed a single-point, targeted disease screen of crabs in the Prince of Wales Dock, Swansea Bay (Wales UK) in July 2021. Using phase contrast microscopy as a primary diagnostic tool, we identified haemolymph borne *Hematodinium* sp. in~16% of crabs, which is in line with previous observations of up to 20% crabs affected at this site during the summer period (June, July, August [6]). Next, we profiled extracellular vesicles and deiminated/citrullinated protein signatures in shore crab haemolymph with patent *Hematodinium* sp. infection and compared those to *Hematodinium*-negative crabs. Broadly, we found fewer systemic EVs in parasitized crabs than healthy ones, coinciding with a shift in modal size to smaller EVs, and differences in the number and composition of deiminated/citrullinated proteins in total haemolymph. Dysregulated EV release in the haemolymph of parasitized crabs can be interpreted as an attempt of *Hematodinium* to compromise the host by disrupting immune cellular (haemocyte) communication/signalling. This is the first study to report EVs in *C. maenas*, where EV profiles were CD63 and Flotillin-1 positive, with heterogeneity in size from~30–500 nm according to NTA profiles, while TEM showed the majority of EVs at a smaller size (≤50 nm); hence some of the NTA measurements are attributed to EV aggregation. A similar observation was made for EVs from lobster and horseshoe crab haemolymph, albeit those were mainly CD63 positive (marker of small EVs, “exosomes” [39,40]), indicating some species-specific differences. We assessed crab haemolymph for a putative PAD homologue via cross-reaction with a human PAD2 antibody, identifying a positive band at the expected size for a PAD (~75 kDa). This is likely cross-reacting with a putative ADI of haemolymph endobionts, according to previous studies on horizontal PAD transfer from cyanobacteria to Chordata and identifying the absence of PAD orthologues in echinoderms, while ADI have been confirmed in a range of bacteria and parasites [33,35,37,38,42,47]. We found no protein sequence for *Hematodinium* sp. ADIs in the available NCBI databases, but an ADI from another dinoflagellate (*Symbiodinium microadriaticum*; OLQ00671.1) was present, and so we aligned the sequence with human PAD isozymes 1–6 (NP_037490.2; NP_031391.2; NP_057317.2; NP_036519.2; NP_997304.3) alongside ADIs from *Giardia intestinalis* (AAC06116.1), a cyanobacterium (*Leptolyngbyaceae* sp. JSC-12; EKQ66906.1) and a teleost fish PAD (*Dicentrarchus labrax*; CBN80708.1) using the ClustalOmega tool (https://www.ebi.ac.uk/Tools/msa/clustalo/). The dinoflagellate ADI grouped closest with the cyanobacterium and *Giardia* ADIs, followed by human PAD6 and PAD2 (Supplementary Figure S1). Hence, citrullinated/deiminated proteins identified here in crab haemolymph are most likely modulated via host-pathogen or host-symbiont interactions.

*Hematodinium* spp. have long been considered agents of host immune suppression, thereby making decapod crustaceans more susceptible to co-infections (e.g [48,49]). Recently, we contested this assertion, and demonstrated that shore crabs from two distinct areas in Swansea Bay (Wales, UK) where *Hematodinium* sp. is enzootic, are no more likely to contract additional parasites/pathogens than *Hematodinium*-free crabs [7]. We stated that *Hematodinium* sp. is a candidate immune evader in shore crabs based on two experimental outcomes: **(1)** haemocytes visible in histological sections (gills and hepatopancreas) of crabs responded to the presence of various microparasites (e.g. yeast-like fungi), macroparasites (e.g. invasive roots of *Sacculina carcini*) and damaged host tissue (e.g. necrotic tubules) regardless of *Hematodinium* sp. presence/absence, and **(2)** haemocytes isolated from healthy crabs and exposed *in vitro* to *Hematodinium* sp. isolated from donor (diseased) crabs did not display typical signs of cell-derived immunity (no degranulation, phagocytosis, or encapsulation [7]). Conversely, in gazami crabs (*P. trituberculatus*) infected with *H. perezi* under laboratory conditions, there is evidence that the host does respond to the dinoflagellate at the mRNA and protein levels – changes in candidate immune gene/protein expression, such as, pro-phenoloxidase or miRNAs [31,32,48] – yet findings are inconsistent. In Li et al. [48], enzyme activities (phenoloxidase, acid and alkaline phosphatases) in the hepatopancreas of *H. perezi* infected *P. trituberculatus* were elevated over 6–192 hours post infection when compared to control crabs, whereas in Li et al. [31] the pro-phenoloxidase activation pathway was significantly downregulated (revealed by multi-omics techniques) as was haemocyte phenoloxidase enzyme activity over the same time frame. Despite some discrepancies among studies, *Hematodinium* spp. take advantage of an altered (or dysfunctional) immune system.

Unique to *Hematodinium*-positive shore crabs, the proteins actin, Down syndrome cell adhesion molecule (DSCAM) and nitric oxide (NO) synthase were identified as deiminated. Actin (cytoskeleton) regulation is necessary for capsule-forming haemocytes in invertebrate systems to adhere to parasitoids or compromised “self” tissues, and, is crucial for vesicle formation/release [50]. The modulation of actin can also disrupt phagosome formation. Out of all the immune-related pathways investigated in *Hematodinium-*infected *P. trituberculatus* by Li et al [31], “regulation of the actin cytoskeleton” and “focal adhesion” were the two most enriched (up to 14-fold). As such, *Hematodinium*-driven post-translational modification could be a mechanism of immune interference.

In earlier work, Li et al [51] described a link between NO synthase and anti-*Hematodinium* responses in *P. trituberculatus*. The haemocytes and hepatopancreases contained high levels of constitutive NO synthase (EC 1.14.13.49.), with both tissues displaying significant increases in gene expression (mRNA) and enzymatic activity when challenged with *H. perezi*. Modulating a host’s NO synthase may be a common strategy among these parasitic dinoflagellates, via deimination/citrullination, and as further evidenced in our *Hematodinium*–shore crab pathosystem. Of course, the catalytic mechanism that NO synthase uses to generate nitric oxide involves the conversion of _L_-arginine to _L_-citrulline. An entirely novel observation is the apparent targeting of DSCAM by *Hematodinium* sp. – a gene with the capacity to yield alternate splice variants (protein isoforms), with multifunctional roles in invertebrate metabolism and immunity (e.g. > 30,000 predicted isoforms in Chinese mitten crabs, *Eriocheir sinensis* [52]). Further modification via deimination/citrullination identified here, would expand the moonlighting abilities of DSCAM. In many arthropods, interfering with DSCAM either by directed mutation or RNAi leads to reduced capacity of haemocytes to recognize and bind to pathogens (e.g. bacteria), as well as carry out phagocytosis [53]. Based on the deiminated proteins detected in *Hematodinium*-positive shore crabs, we can speculate that haemocyte-haemocyte communication is impaired – also reflected in the altered EV profiles – and that haemocytes are prevented from adhering to, and circumscribing the spread of, *Hematodinium* sp. Furthermore, in *Hematodinium*-negative crabs the actin-binding protein, eukaryotic elongation factor 1A, was deiminated, which also suggests that actin is a key player in disease outcomes. Interestingly, we found indirect evidence of PAD-associated extracellular trap formation (ETosis) via histone H3 citrullination in both *Hematodinium*-positive and negative crabs. Haemocyte-mediated ETosis is the functional equivalent to NETosis (performed by neutrophils) and is an ancient cellular defence mechanism considered a pre-requisite for bacterial encapsulation (or ensheathment) in *C. maenas* (at least in the gills [54]. The cross reactivity of the CitH3 antibody with proteins in the crab haemolymph as well as the detection of deiminated/citrullinated histone H3 in the mass spectrometry profiles suggest a more nuanced relationship between haemocytes and *Hematodinium* sp.; the strong signal at the lower positive band for citH3 in parasitized crabs is evidence of increased ETosis, but we will not speculate further.

Seven deiminated proteins common among parasitized and control crabs were identified (e.g. hemocyanin subunit b; Table 1), with an additional five being unique to *Hematodinium*-free crabs, including the antimicrobial peptide carcinin (a member of the crustin AMPs). Carcinin is a potent antibacterial peptide distributed among diverse tissue types in *C. maenas* [29,55], and is a likely candidate for deimination by symbiotic residents. The haemolymph of *C. maenas* (like many invertebrates) is not always sterile, routinely hosting endobiotic bacteria 10-1000s per mL [6], in addition to viruses and other microeukaryotes [3,56]. Symbiont-mediated deimination of haemolymph factors like carcinin and hemocyanin (having roles in immunity and respiration [57], could be, to a certain extent, part of the balance between host and residents, also aligning with previous studies highlighting possible citrullination of host proteins by symbionts [38,39–41,42,47]. Disturbance to this balance would induce host-reactivity and/or pathobiont emergence from the haemolymph microbiome. Beyond immunity, deiminated proteins in shore crab haemolymph were associated with diverse physiological tasks: ecdysis (Corazonin receptor), central metabolism (glyceraldehyde-3-phosphate dehydrogenase), the cell stress response (heat shock protein 70) and oogenesis (vitellogenin). These data complement an increasing body of work characterizing the interplay between marine invertebrates and their symbionts [38,39–41,42].

### Concluding remarks

This is the first report of EV profiles and deiminated proteins in shore crabs, as well as investigating these parameters in relation to disease caused by parasitic dinoflagellates. *Hematodinium* spp. are known for their ability to not elicit cellular (haemocyte) immune defences of shore crabs, including phagocytosis and encapsulation, and evidence presented here indicate that one such mechanism is through disruption of EV biogenesis (a proxy for cellular communication) – reducing the number and size of systemic EVs in the haemolymph. Furthermore, parasitized crabs showed differences in protein deimination/citrullination signatures associated with haemocyte adhesion (notably DSCAM), compared to *Hematodinium*-free crabs. These findings support a role for pathogens and/or commensals in modulating host immune responses via deimination.

## Supplementary Material

Supplemental Material

## Data Availability

The authors confirm that the data supporting the findings of this study are available within the article and its supplementary materials.
