# Supplementary material for: Extracellular vesicle signatures and protein citrullination are modified in shore crabs (Carcinus maenas) infected with Hematodinium sp
Source: Virulence. 2023 Mar 2;14(1):2180932. doi: 10.1080/21505594.2023.2180932 (PMC9988307; doi:10.1080/21505594.2023.2180932)
Supplement: Supplemental Material [file KVIR_A_2180932_SM4632.docx]

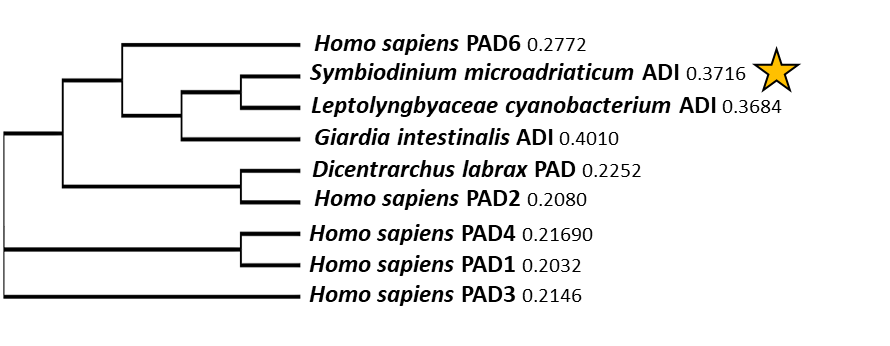


**Supplementary Figure 1: Gene tree of peptidylarginine deiminases and arginine deiminases (dinoflagellate ADI tree).** ADI from a representative dinoflagellate, *Symbiodinium microadraticum* (yellow star) groups with ADI from cyanobacteria and Giardia, and shows closest homology to human PAD6, followed by PAD2 and teleost PAD (*Dicentrarchus labrax* as a representative species). The alignment and tree were constructed in ClustalOmega (https://www.ebi.ac.uk/Tools/msa/clustalo/); sequences used: OLQ00671.1; NP_037490.2; NP_031391.2; NP_057317.2; NP_036519.2; NP_997304.3; AAC06116.1; EKQ66906.1; CBN80708.1. Measure of support for each node is indicated by the numbers next to the species name.

**Supplementary Table 1 Biometric data for selected *Hematodinium*-positive and negative crabs**

| Crab identifier^1^ | *Hematodinium* sp. microscopy^2^ and PCR^3^ | | Ratio of *Hematodinium* to haemocytes | Carapace width  (mm) | Sex | Fouling^5^ | Limb loss | Haemolymph appearance | CFUs per mL^4^ | Sample pool |
| --- | --- | --- | --- | --- | --- | --- | --- | --- | --- | --- |
| No. 5 | | **Yes** | 1: 9 | 56 | Male | No | No | Milky | 0 | A |
| No. 12 | | **Yes** | 1: 18 | 60 | Female | Yes | No | Normal | 170 | B |
| No. 30 | | **Yes** | 1:17 | 50 | Female | No | No | Normal | 0 | B |
| No. 32 | | **Yes** | 1: 29 | 59 | Female | No | No | Milky | 0 | B |
| No. 38 | | **Yes** | 1: 33 | 59 | Male | No | No | Normal | 90 | A |
| No. 41 | | **Yes** | 1: 28 | 48 | Male | No | No | Milky | 0 | A |
| No. 7 | | No | N/A | 62 | Female | No | No | Normal | 190 | D |
| No. 14 | | No | N/A | 55 | Female | No | No | Normal | 0 | D |
| No. 23 | | No | N/A | 57 | Male | No | No | Normal | 20 | C |
| No. 36 | | No | N/A | 63 | Male | Yes | No | Normal | 110 | C |
| No. 44 | | No | N/A | 53 | Male | No | No | Normal | 0 | C |
| No. 53 | | No | N/A | 50 | Female | No | No | Normal | 0 | D |

1. In total, 58 crabs were screened for *Hematodinium* sp.
2. Inspection of fresh haemolymph under phase contrast settings
3. Amplicon (187 bp) generated from haemolymph DNA using *Hematodinium*-specific primers
4. Number of cultivable bacteria per ml of crab haemolymph
5. Presence of epibionts on the exoskeleton surface
